# *catena*-Poly[[[diaqua­copper(II)]-bis­(μ_2_-di-4-pyridyl disulfide-κ^2^ *N*:*N*′)] bis­(hydrogen phthalate) monohydrate]

**DOI:** 10.1107/S1600536810001716

**Published:** 2010-01-20

**Authors:** Hong-Lin Zhu, Jie Zhang, Jian-Li Lin

**Affiliations:** a State Key Laboratory Base of Novel Functional Materials and Preparation Science, Center of Applied Solid State Chemistry Research, Ningbo University, Ningbo, Zhejiang 315211, People’s Republic of China

## Abstract

The asymmetric unit of the title compound, {[Cu(C_10_H_8_N_2_S_2_)_2_(H_2_O)_2_](C_8_H_5_O_4_)_2_·H_2_O}_*n*_, contains one Cu^II^ ion, two bridging di-4-pyridyl disulfide (4-DPDS) ligands of the same chirality, two coordinating water mol­ecules, two hydrogen phthalate anions and one uncoordinated water mol­ecule. The polymeric structure consists of two types of polymeric chains, each composed from repeated chiral rhomboids. The Cu^II^ ions adopt a distorted octa­hedral coordination geometry and are coordinated by four pyridine N atoms and two water O atoms. The coordinated water mol­ecules and hydrogen phthalate anions are located between the repeated rhomboidal chains, and form hydrogen bonds with the coordinated water mol­ecules.

## Related literature

For general background to 4,4′-dipyridyldisulfide, see Horikoshi & Mochida (2006 ▶). For coordination complexes with the title ligand, see: Manna *et al.* (2005 ▶, 2007 ▶); Luo *et al.* (2003 ▶).
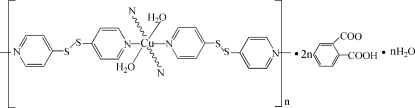


## Experimental

### 

#### Crystal data

[Cu(C_10_H_8_N_2_S_2_)_2_(H_2_O)_2_](C_8_H_5_O_4_)_2_·H_2_O
*M* *_r_* = 888.44
Orthorhombic, 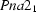

*a* = 20.253 (4) Å
*b* = 10.732 (2) Å
*c* = 17.228 (3) Å
*V* = 3744.6 (13) Å^3^
*Z* = 4
Mo *K*α radiation
μ = 0.88 mm^−1^
*T* = 295 K
0.40 × 0.13 × 0.12 mm

#### Data collection

Rigaku R-AXIS RAPID diffractometer
Absorption correction: multi-scan (*ABSCOR*; Higashi, 1995 ▶) *T*_min_ = 0.870, *T*_max_ = 0.901
34124 measured reflections
8513 independent reflections
5968 reflections with *I* > 2σ(*I*)
*R*_int_ = 0.077

#### Refinement

*R*[*F* ^2^ > 2σ(*F* ^2^)] = 0.045
*wR*(*F* ^2^) = 0.081
*S* = 1.02
8513 reflections
506 parameters
1 restraint
H-atom parameters constrained
Δρ_max_ = 0.25 e Å^−3^
Δρ_min_ = −0.32 e Å^−3^
Absolute structure: Flack (1983 ▶), 4088 Friedel pairs
Flack parameter: 0.00 (7)

### 

Data collection: *RAPID-AUTO* (Rigaku, 1998 ▶); cell refinement: *RAPID-AUTO*; data reduction: *CrystalStructure* (Rigaku/MSC, 2004 ▶); program(s) used to solve structure: *SHELXS97* (Sheldrick, 2008 ▶); program(s) used to refine structure: *SHELXL97* (Sheldrick, 2008 ▶); molecular graphics: *SHELXTL* (Sheldrick, 2008 ▶); software used to prepare material for publication: *SHELXL97*.

## Supplementary Material

Crystal structure: contains datablocks global, I. DOI: 10.1107/S1600536810001716/cv2686sup1.cif

Structure factors: contains datablocks I. DOI: 10.1107/S1600536810001716/cv2686Isup2.hkl

Additional supplementary materials:  crystallographic information; 3D view; checkCIF report

## Figures and Tables

**Table 1:** Hydrogen-bond geometry (Å, °)

*D*—H⋯*A*	*D*—H	H⋯*A*	*D*⋯*A*	*D*—H⋯*A*
O1—H*W*1⋯O7	0.81	2.51	3.118 (6)	133
O1—H*W*2⋯O3^i^	0.81	1.92	2.658 (4)	153
O2—H2*C*⋯O7^ii^	0.75	2.13	2.878 (4)	174
O2—H2*D*⋯O9	0.80	2.05	2.841 (4)	171
O3—H3*C*⋯O4^ii^	0.81	2.02	2.800 (4)	163
O3—H3*D*⋯O11	0.76	2.03	2.784 (5)	172
O5—H5*C*⋯O6	0.85	1.51	2.358 (5)	178
O8—H8*C*⋯O11	0.87	1.50	2.367 (4)	178

Symmetry codes: (i) 
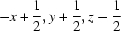
; (ii) 
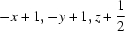
.

**Fig. 1.**
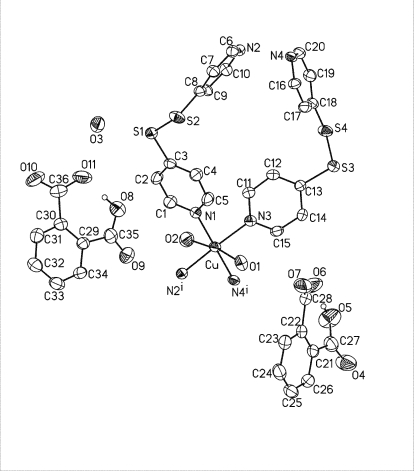
The content of asymmetric unit showing the atomic numbering and 45% probability dispalcement ellipsoids [symmetry code: (i) x, y + 1, z]. Most of H-atoms omitted for clarity

**Fig. 2.**
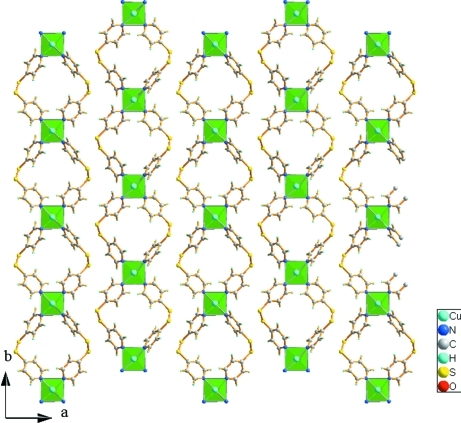
A portion of the crystal packing viewed along axis c and showing the polymeric chains composed from the CuII ions and 4,4'-DPDS ligands. Anions and lattice water molecules were omitted for clarity.

**Fig. 3.**
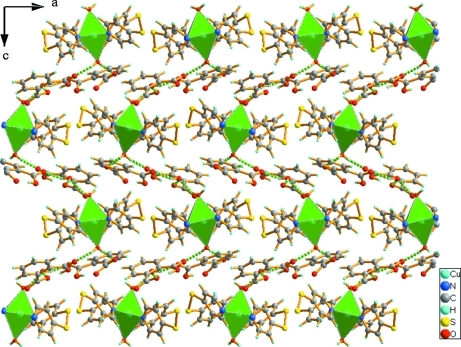
A portion of the crystal packing viewed along axis b and showing O—H···O hydrogen bonds as dashed lines.

## supplementary crystallographic information

### Comment

Over past few years, the 4,4'-dipyridyldisulfide has received considerable attention due to both its conformational flexibility and axial chirality (Horikoshi & Mochida, 2006). A large number of metal coordination networks have been reported by using these ligands only or in combination with suitable anions (Manna *et al.*, 2005, 2007; Luo *et al.*, 2003). In this contribution, we report the synthesis and crystal structure of the title compound.

The asymmetric unit of the title compound, {[Cu(4-DPDS)_2_(H_2_O)_2_].2(C_8_H_5_O_4_)^-^.H_2_O}_n_ (4-DPDS = 4,4'-dipyridinedisulfide),contains one Cu^II^ ion, two bridging 4-DPDS ligands of the same chirality, two coordinating water molecules, two hydrogen phthalate anions and one lattice water molecule (Fig. 1). The copper atoms are each coordinated by four pyridine nitrogen atoms and two aqua ligands to complete an elongated octahedral CuN_4_O_2_ chromophore of "4 + 2" coordination type due to Jahn-Teller effect. The equatorial positions are occupied by four N toms of four 4-DPDS ligands, and the axial ones by two aqua O atoms. The Cu—O distances of 2.513 (3) Å and 2.438 (3) Å are significantly larger than those to the nitrogen atoms (Cu—N = 2.031 (3)–2.054 (3) Å), indicating a weak coordination capability of the aqua ligand. The *cis* and *trans*  N—Cu—N angles fall in the regions 88.97 (9)–90.99 (9)° and 173.34 (13)–176.78 (12)°, respectively, exhibiting small deviation from the corresponding values for a regular geometry. The bond lengths (with  the Cu atoms) are all within the normal ranges (Manna *et al.*, 2007).

The Cu atoms are bridged by four 4-DPDS ligands to form one-dimensional double–stranded chains extending in the [010] direction. The one-dimensional chains with respect to the neighbour are close-packed in ···ABAB··· sequence (Fig. 2). Despite the Cu—Cu distance spanned by the two 4-DPDS are 10.732 (2) Å, but no similar mesoporous structure form, because the H*L*^-^ anions and lattice H_2_O molecule reside in cavities in the one-dimensional chain metallacycle. The H*L*^-^ anions play a role in balancing in charge, the carboxylic –COOH groups favor formation of strong intramolecular hydrogen bond to carboxylate O6 atom and O11 atom, and the lattice H_2_O molecule form hydrogen bonds to two H*L*^-^ ainions. The distance of S—S between adjacent chains is 5.17 (1) Å, which is much greater than van der waals distance (3.7 Å), shows that there is no S—S weak interaction.The chains are linked *via* those interchain hydrogen bonds between the aqua ligand and the carboxylate atoms (Table 1) into two-dimensional layers (Fig. 3).

### Experimental

Dropwise addition of 0.5 ml 1.0 *M* NaOH to a aqueous solution of Cu(NO_3_)_2_.3H_2_O (0.0603 g, 0.25 mmol) in 4 ml H_2_O produced the blue precipitate, which was then centrifuged and washed with double-distilled water four times. The precipitate was subsequently moved to a stirred suspension of phthalic acid (0.0510 g, 0.25 mmol) and DPDS (4,4'-dipyridinedisulfide) (0.0575 g, 0.25 mmol) in 30 ml hot water. The mixture was further stirred for 30 min and the insoluble solid was filtere off. The colourless filtrate was allowed to stand at the room temperature. Slow evaporation for about a month afforded a small amount of blue block crystals.

### Refinement

H atoms bonded to C atoms were geometrically positioned and were refined using a riding model, with *U*_iso_(H) = 1.2 *U*_eq_(C). H atoms attached to O atoms were found in a difference Fourier synthesis and were refined using a riding model, with the O—H distances fixed as initially found and with *U*_iso_(H) values set at 1.2 *U*eq(O).

### Crystal data

**Table:**

[Cu(C_10_H_8_N_2_S_2_)_2_(H_2_O)_2_](C_8_H_5_O_4_)_2_·H_2_O	*F*(000) = 1828
*M**_r_* = 888.44	*D*_x_ = 1.576 Mg m^−^^3^
Orthorhombic, *P**n**a*2_1_	Mo *K*α radiation, λ = 0.71073 Å
Hall symbol: P 2c -2n	Cell parameters from 34124 reflections
*a* = 20.253 (4) Å	θ = 3.0–27.5°
*b* = 10.732 (2) Å	µ = 0.88 mm^−^^1^
*c* = 17.228 (3) Å	*T* = 295 K
*V* = 3744.6 (13) Å^3^	Chip, blue
*Z* = 4	0.40 × 0.13 × 0.12 mm

### Data collection

**Table:**

Rigaku R-AXIS RAPID diffractometer	8513 independent reflections
Radiation source: fine-focus sealed tube	5968 reflections with *I* > 2σ(*I*)
graphite	*R*_int_ = 0.077
Detector resolution: 0 pixels mm^-1^	θ_max_ = 27.5°, θ_min_ = 3.0°
ω scan	*h* = −26→25
Absorption correction: multi-scan (*ABSCOR*; Higashi, 1995)	*k* = −13→12
*T*_min_ = 0.870, *T*_max_ = 0.901	*l* = −22→22
34124 measured reflections	

### Refinement

**Table:**

Refinement on *F*^2^	Secondary atom site location: difference Fourier map
Least-squares matrix: full	Hydrogen site location: inferred from neighbouring sites
*R*[*F*^2^ > 2σ(*F*^2^)] = 0.045	H-atom parameters constrained
*wR*(*F*^2^) = 0.081	*w* = 1/[σ^2^(*F*_o_^2^) + (0.0297*P*)^2^ + 0.2143*P*] where *P* = (*F*_o_^2^ + 2*F*_c_^2^)/3
*S* = 1.02	(Δ/σ)_max_ = 0.004
8513 reflections	Δρ_max_ = 0.25 e Å^−^^3^
506 parameters	Δρ_min_ = −0.31 e Å^−^^3^
1 restraint	Absolute structure: Flack (1983), 4088 Friedel pairs
Primary atom site location: structure-invariant direct methods	Flack parameter: 0.00 (7)

### Special details

**Table:**

Geometry. All e.s.d.'s (except the e.s.d. in the dihedral angle between two l.s. planes) are estimated using the full covariance matrix. The cell e.s.d.'s are taken into account individually in the estimation of e.s.d.'s in distances, angles and torsion angles; correlations between e.s.d.'s in cell parameters are only used when they are defined by crystal symmetry. An approximate (isotropic) treatment of cell e.s.d.'s is used for estimating e.s.d.'s involving l.s. planes.
Refinement. Refinement of *F*^2^ against ALL reflections. The weighted *R*-factor *wR* and goodness of fit *S* are based on *F*^2^, conventional *R*-factors *R* are based on *F*, with *F* set to zero for negative *F*^2^. The threshold expression of *F*^2^ > σ(*F*^2^) is used only for calculating *R*-factors(gt) *etc*. and is not relevant to the choice of reflections for refinement. *R*-factors based on *F*^2^ are statistically about twice as large as those based on *F*, and *R*- factors based on ALL data will be even larger.

### Fractional atomic coordinates and isotropic or equivalent isotropic displacement parameters (Å^2^)

**Table:**

	*x*	*y*	*z*	*U*_iso_*/*U*_eq_	
Cu	0.419363 (19)	0.57242 (3)	0.25910 (3)	0.03533 (11)	
N1	0.35058 (13)	0.4380 (2)	0.27778 (17)	0.0352 (7)	
C1	0.32045 (18)	0.4283 (3)	0.3466 (2)	0.0414 (9)	
H1A	0.3299	0.4871	0.3846	0.050*	
C2	0.27606 (18)	0.3352 (3)	0.3638 (2)	0.0423 (9)	
H2A	0.2559	0.3322	0.4123	0.051*	
C3	0.26177 (16)	0.2469 (3)	0.3089 (2)	0.0317 (8)	
C4	0.29200 (18)	0.2565 (3)	0.2372 (2)	0.0422 (9)	
H4A	0.2833	0.1987	0.1983	0.051*	
C5	0.33519 (18)	0.3531 (3)	0.2245 (2)	0.0459 (10)	
H5A	0.3548	0.3596	0.1758	0.055*	
S1	0.20343 (4)	0.13124 (8)	0.33513 (6)	0.0442 (2)	
S2	0.19610 (4)	0.01528 (8)	0.24265 (6)	0.0455 (3)	
C6	0.29384 (16)	−0.3035 (3)	0.2225 (2)	0.0358 (8)	
H6A	0.2874	−0.3766	0.1945	0.043*	
C7	0.24750 (15)	−0.2108 (3)	0.2161 (2)	0.0359 (9)	
H7A	0.2110	−0.2204	0.1839	0.043*	
C8	0.25621 (15)	−0.1028 (3)	0.2586 (3)	0.0337 (8)	
C9	0.31050 (16)	−0.0918 (3)	0.3058 (2)	0.0348 (8)	
H9A	0.3171	−0.0205	0.3356	0.042*	
C10	0.35480 (16)	−0.1884 (3)	0.3081 (2)	0.0343 (8)	
H10A	0.3917	−0.1802	0.3398	0.041*	
N2	0.34792 (11)	−0.2942 (2)	0.26704 (19)	0.0323 (6)	
N3	0.48781 (13)	0.4353 (2)	0.23799 (18)	0.0375 (7)	
C11	0.49242 (17)	0.3287 (3)	0.2780 (2)	0.0442 (10)	
H11A	0.4667	0.3192	0.3224	0.053*	
C12	0.53371 (16)	0.2320 (3)	0.2564 (3)	0.0433 (9)	
H12A	0.5361	0.1603	0.2866	0.052*	
C13	0.57126 (15)	0.2425 (3)	0.1901 (2)	0.0338 (8)	
C14	0.56681 (15)	0.3531 (3)	0.1478 (2)	0.0350 (8)	
H14A	0.5912	0.3642	0.1026	0.042*	
C15	0.52524 (16)	0.4454 (3)	0.1747 (2)	0.0385 (9)	
H15A	0.5232	0.5195	0.1468	0.046*	
S3	0.62621 (4)	0.12937 (8)	0.15263 (6)	0.0439 (2)	
S4	0.64454 (4)	0.01289 (8)	0.24275 (6)	0.0450 (3)	
C16	0.48282 (16)	−0.1984 (3)	0.1973 (2)	0.0348 (8)	
H16A	0.4458	−0.1968	0.1653	0.042*	
C17	0.52745 (17)	−0.1020 (3)	0.1922 (2)	0.0406 (9)	
H17A	0.5204	−0.0364	0.1580	0.049*	
C18	0.58314 (16)	−0.1044 (3)	0.2391 (2)	0.0350 (9)	
C19	0.59177 (16)	−0.2049 (3)	0.2884 (2)	0.0384 (9)	
H19A	0.6290	−0.2096	0.3200	0.046*	
C20	0.54505 (16)	−0.2978 (3)	0.2905 (2)	0.0380 (9)	
H20A	0.5515	−0.3651	0.3236	0.046*	
N4	0.49040 (12)	−0.2948 (2)	0.24643 (19)	0.0328 (7)	
O1	0.40118 (12)	0.5777 (2)	0.11927 (16)	0.0486 (7)	
HW1	0.4278	0.5412	0.0925	0.073*	
HW2	0.3717	0.6234	0.1060	0.073*	
O2	0.43965 (13)	0.6061 (2)	0.40149 (16)	0.0542 (7)	
H2C	0.4576	0.5521	0.4193	0.081*	
H2D	0.4114	0.6375	0.4277	0.081*	
O3	0.18807 (13)	0.1902 (3)	0.53050 (18)	0.0614 (8)	
H3C	0.2246	0.1603	0.5294	0.092*	
H3D	0.1909	0.2582	0.5422	0.092*	
O4	0.69101 (15)	0.9223 (3)	−0.0056 (3)	0.0932 (13)	
O5	0.65614 (15)	0.7585 (4)	0.0559 (2)	0.0996 (13)	
H5C	0.6245	0.7070	0.0509	0.149*	
C27	0.6463 (2)	0.8540 (4)	0.0128 (3)	0.0566 (11)	
C21	0.57687 (18)	0.8814 (3)	−0.0151 (2)	0.0421 (9)	
C22	0.52332 (18)	0.7981 (3)	−0.0218 (2)	0.0426 (9)	
C23	0.46327 (19)	0.8428 (4)	−0.0462 (2)	0.0520 (11)	
H23A	0.4277	0.7885	−0.0499	0.062*	
C24	0.4545 (2)	0.9676 (4)	−0.0656 (3)	0.0579 (11)	
H24A	0.4133	0.9961	−0.0816	0.069*	
C25	0.5070 (2)	1.0483 (4)	−0.0610 (3)	0.0547 (11)	
H25A	0.5016	1.1318	−0.0740	0.066*	
C26	0.5675 (2)	1.0050 (3)	−0.0370 (2)	0.0499 (10)	
H26A	0.6031	1.0597	−0.0354	0.060*	
C28	0.5238 (3)	0.6591 (4)	−0.0040 (3)	0.0680 (15)	
O6	0.56712 (19)	0.6184 (4)	0.0433 (3)	0.1161 (17)	
O7	0.48227 (18)	0.5928 (3)	−0.0343 (3)	0.0952 (13)	
O8	0.30452 (14)	0.5485 (3)	0.52104 (19)	0.0687 (9)	
H8C	0.2702	0.5089	0.5380	0.103*	
O9	0.33393 (14)	0.7296 (3)	0.4778 (2)	0.0710 (9)	
C35	0.2903 (2)	0.6610 (4)	0.5027 (2)	0.0478 (10)	
C29	0.21993 (17)	0.7079 (3)	0.5099 (2)	0.0379 (8)	
C30	0.16576 (17)	0.6471 (3)	0.5444 (2)	0.0377 (9)	
C31	0.10515 (19)	0.7074 (4)	0.5451 (2)	0.0482 (10)	
H31A	0.0696	0.6688	0.5693	0.058*	
C32	0.0956 (2)	0.8229 (3)	0.5111 (3)	0.0527 (11)	
H32A	0.0540	0.8596	0.5105	0.063*	
C33	0.1488 (2)	0.8823 (4)	0.4782 (3)	0.0539 (11)	
H33A	0.1436	0.9605	0.4558	0.065*	
C34	0.2096 (2)	0.8260 (3)	0.4787 (2)	0.0490 (10)	
H34A	0.2453	0.8683	0.4573	0.059*	
C36	0.1630 (2)	0.5172 (4)	0.5815 (3)	0.0543 (11)	
O10	0.11684 (16)	0.4905 (3)	0.6230 (2)	0.0873 (11)	
O11	0.20996 (16)	0.4401 (3)	0.5653 (2)	0.0775 (10)	

### Atomic displacement parameters (Å^2^)

**Table:**

	*U*^11^	*U*^22^	*U*^33^	*U*^12^	*U*^13^	*U*^23^
Cu	0.03143 (18)	0.02371 (17)	0.0508 (3)	−0.00042 (18)	0.0054 (2)	0.0016 (2)
N1	0.0402 (15)	0.0293 (14)	0.036 (2)	0.0017 (13)	0.0073 (14)	0.0007 (14)
C1	0.048 (2)	0.0368 (19)	0.039 (2)	−0.0102 (18)	0.0013 (18)	−0.0095 (18)
C2	0.053 (2)	0.0400 (19)	0.034 (2)	−0.0096 (18)	0.0133 (18)	−0.0055 (17)
C3	0.0290 (18)	0.0254 (16)	0.041 (2)	0.0015 (14)	−0.0044 (16)	0.0034 (16)
C4	0.057 (2)	0.0356 (18)	0.034 (2)	−0.0093 (16)	0.0065 (19)	−0.0097 (16)
C5	0.056 (2)	0.044 (2)	0.037 (2)	−0.0084 (19)	0.0142 (19)	−0.0073 (18)
S1	0.0399 (5)	0.0312 (4)	0.0614 (7)	−0.0034 (4)	0.0134 (5)	0.0008 (5)
S2	0.0371 (5)	0.0307 (4)	0.0687 (8)	0.0029 (4)	−0.0144 (5)	−0.0018 (5)
C6	0.0362 (19)	0.0324 (18)	0.039 (2)	−0.0068 (15)	0.0018 (17)	−0.0039 (16)
C7	0.0314 (18)	0.0315 (17)	0.045 (2)	−0.0046 (15)	−0.0047 (16)	−0.0037 (16)
C8	0.0334 (16)	0.0278 (16)	0.040 (2)	−0.0037 (14)	0.0005 (19)	0.0023 (18)
C9	0.0370 (19)	0.0266 (17)	0.041 (2)	0.0004 (15)	−0.0051 (16)	−0.0080 (16)
C10	0.0309 (18)	0.0353 (19)	0.037 (2)	−0.0018 (16)	−0.0049 (16)	−0.0058 (17)
N2	0.0307 (14)	0.0301 (13)	0.0362 (18)	−0.0018 (11)	0.0018 (14)	0.0007 (14)
N3	0.0365 (15)	0.0293 (14)	0.047 (2)	−0.0005 (12)	0.0062 (14)	0.0027 (14)
C11	0.052 (2)	0.0352 (18)	0.046 (3)	0.0056 (17)	0.0127 (19)	0.0110 (17)
C12	0.050 (2)	0.0301 (16)	0.050 (2)	0.0062 (15)	0.009 (2)	0.012 (2)
C13	0.0305 (18)	0.0291 (16)	0.042 (2)	−0.0005 (14)	0.0045 (18)	0.0031 (16)
C14	0.0297 (17)	0.0367 (18)	0.039 (2)	0.0001 (14)	0.0087 (16)	0.0028 (16)
C15	0.0366 (19)	0.0316 (18)	0.047 (3)	−0.0022 (15)	0.0019 (18)	0.0120 (17)
S3	0.0418 (5)	0.0314 (4)	0.0584 (7)	0.0026 (4)	0.0124 (5)	0.0026 (4)
S4	0.0316 (4)	0.0300 (4)	0.0734 (8)	−0.0020 (3)	−0.0050 (5)	0.0051 (5)
C16	0.0304 (18)	0.0295 (16)	0.044 (2)	−0.0040 (14)	−0.0013 (17)	0.0054 (17)
C17	0.041 (2)	0.0324 (18)	0.048 (2)	0.0023 (16)	0.0015 (18)	0.0096 (17)
C18	0.0297 (15)	0.0240 (14)	0.051 (3)	0.0028 (15)	0.0039 (17)	−0.0019 (15)
C19	0.0311 (18)	0.0360 (18)	0.048 (2)	0.0010 (15)	−0.0037 (16)	−0.0010 (17)
C20	0.0315 (18)	0.0322 (18)	0.050 (3)	0.0048 (16)	0.0016 (17)	0.0061 (16)
N4	0.0312 (13)	0.0237 (12)	0.044 (2)	0.0039 (11)	0.0003 (15)	0.0040 (14)
O1	0.0451 (14)	0.0537 (16)	0.0470 (17)	0.0114 (12)	0.0003 (13)	−0.0071 (14)
O2	0.0470 (15)	0.0574 (16)	0.058 (2)	0.0032 (14)	0.0009 (14)	0.0067 (14)
O3	0.0564 (17)	0.0610 (17)	0.067 (2)	−0.0052 (15)	0.0131 (15)	−0.0041 (16)
O4	0.0556 (19)	0.0593 (19)	0.165 (4)	−0.0057 (16)	−0.005 (2)	0.012 (2)
O5	0.079 (2)	0.122 (3)	0.098 (3)	0.011 (2)	−0.022 (2)	0.058 (2)
C27	0.055 (3)	0.056 (3)	0.059 (3)	0.013 (2)	0.002 (2)	0.002 (2)
C21	0.052 (2)	0.0399 (19)	0.034 (2)	0.0076 (18)	0.0094 (19)	0.0007 (16)
C22	0.049 (2)	0.040 (2)	0.039 (2)	0.0035 (18)	0.0159 (19)	−0.0022 (18)
C23	0.051 (2)	0.051 (2)	0.054 (3)	−0.001 (2)	0.013 (2)	−0.003 (2)
C24	0.054 (3)	0.061 (3)	0.059 (3)	0.016 (2)	0.006 (2)	−0.003 (2)
C25	0.069 (3)	0.040 (2)	0.055 (3)	0.013 (2)	0.006 (2)	0.002 (2)
C26	0.058 (3)	0.042 (2)	0.050 (3)	−0.0017 (19)	0.013 (2)	0.0001 (19)
C28	0.068 (3)	0.047 (3)	0.089 (4)	0.010 (3)	0.045 (3)	0.014 (3)
O6	0.095 (3)	0.096 (3)	0.158 (4)	0.012 (2)	0.014 (3)	0.083 (3)
O7	0.084 (2)	0.0428 (17)	0.159 (4)	−0.0101 (17)	0.035 (3)	−0.004 (2)
O8	0.0650 (19)	0.0590 (18)	0.082 (2)	0.0120 (15)	0.0089 (17)	0.0084 (17)
O9	0.0537 (18)	0.0671 (19)	0.092 (3)	−0.0054 (16)	0.0205 (17)	0.0079 (18)
C35	0.052 (3)	0.053 (2)	0.038 (2)	0.004 (2)	−0.001 (2)	−0.003 (2)
C29	0.046 (2)	0.0341 (18)	0.034 (2)	−0.0047 (16)	0.0011 (18)	−0.0036 (16)
C30	0.046 (2)	0.0342 (19)	0.032 (2)	−0.0065 (17)	−0.0051 (17)	−0.0059 (16)
C31	0.046 (2)	0.052 (2)	0.047 (3)	−0.0093 (19)	0.0019 (19)	−0.007 (2)
C32	0.055 (3)	0.042 (2)	0.062 (3)	0.003 (2)	−0.003 (2)	−0.008 (2)
C33	0.070 (3)	0.036 (2)	0.055 (3)	0.007 (2)	0.000 (2)	−0.001 (2)
C34	0.062 (3)	0.0351 (19)	0.050 (3)	−0.0053 (19)	0.014 (2)	0.0015 (18)
C36	0.061 (3)	0.043 (2)	0.058 (3)	−0.013 (2)	−0.008 (2)	0.008 (2)
O10	0.084 (2)	0.074 (2)	0.104 (3)	−0.0180 (19)	0.023 (2)	0.036 (2)
O11	0.082 (2)	0.0426 (16)	0.108 (3)	0.0064 (16)	0.002 (2)	0.0206 (18)

### Geometric parameters (Å, °)

**Table:**

Cu—N1	2.031 (3)	C17—H17A	0.9300
Cu—N4^i^	2.037 (2)	C18—C19	1.384 (5)
Cu—N2^i^	2.040 (2)	C19—C20	1.374 (4)
Cu—N3	2.054 (3)	C19—H19A	0.9300
Cu—O1	2.438 (3)	C20—N4	1.343 (4)
Cu—O2	2.513 (3)	C20—H20A	0.9300
N1—C5	1.330 (4)	N4—Cu^ii^	2.037 (2)
N1—C1	1.338 (4)	O1—HW1	0.8101
C1—C2	1.376 (5)	O1—HW2	0.8061
C1—H1A	0.9300	O2—H2C	0.7498
C2—C3	1.370 (5)	O2—H2D	0.8035
C2—H2A	0.9300	O3—H3C	0.8067
C3—C4	1.383 (5)	O3—H3D	0.7598
C3—S1	1.772 (3)	O4—C27	1.207 (5)
C4—C5	1.374 (5)	O5—C27	1.281 (5)
C4—H4A	0.9300	O5—H5C	0.8501
C5—H5A	0.9300	C27—C21	1.516 (5)
S1—S2	2.0271 (15)	C21—C26	1.392 (5)
S2—C8	1.778 (3)	C21—C22	1.411 (5)
C6—N2	1.341 (4)	C22—C23	1.374 (5)
C6—C7	1.372 (4)	C22—C28	1.523 (5)
C6—H6A	0.9300	C23—C24	1.391 (5)
C7—C8	1.382 (4)	C23—H23A	0.9300
C7—H7A	0.9300	C24—C25	1.374 (5)
C8—C9	1.373 (5)	C24—H24A	0.9300
C9—C10	1.372 (4)	C25—C26	1.373 (5)
C9—H9A	0.9300	C25—H25A	0.9300
C10—N2	1.346 (4)	C26—H26A	0.9300
C10—H10A	0.9300	C28—O7	1.219 (6)
N2—Cu^ii^	2.040 (2)	C28—O6	1.275 (6)
N3—C15	1.332 (4)	O8—C35	1.281 (4)
N3—C11	1.339 (4)	O8—H8C	0.8655
C11—C12	1.384 (4)	O9—C35	1.228 (4)
C11—H11A	0.9300	C35—C29	1.516 (5)
C12—C13	1.378 (5)	C29—C34	1.393 (5)
C12—H12A	0.9300	C29—C30	1.408 (5)
C13—C14	1.396 (4)	C30—C31	1.388 (5)
C13—S3	1.769 (3)	C30—C36	1.535 (5)
C14—C15	1.381 (4)	C31—C32	1.385 (5)
C14—H14A	0.9300	C31—H31A	0.9300
C15—H15A	0.9300	C32—C33	1.374 (5)
S3—S4	2.0275 (14)	C32—H32A	0.9300
S4—C18	1.771 (3)	C33—C34	1.372 (5)
C16—N4	1.346 (4)	C33—H33A	0.9300
C16—C17	1.376 (4)	C34—H34A	0.9300
C16—H16A	0.9300	C36—O10	1.211 (5)
C17—C18	1.388 (5)	C36—O11	1.291 (5)
			
N1—Cu—N4^i^	176.78 (12)	N4—C16—H16A	118.6
N1—Cu—N2^i^	90.08 (10)	C17—C16—H16A	118.6
N4^i^—Cu—N2^i^	90.99 (9)	C16—C17—C18	118.9 (3)
N1—Cu—N3	88.97 (9)	C16—C17—H17A	120.6
N4^i^—Cu—N3	90.31 (10)	C18—C17—H17A	120.6
N2^i^—Cu—N3	173.34 (13)	C19—C18—C17	118.3 (3)
N1—Cu—O1	93.99 (10)	C19—C18—S4	116.3 (3)
N4^i^—Cu—O1	89.11 (11)	C17—C18—S4	125.3 (2)
N2^i^—Cu—O1	86.72 (11)	C20—C19—C18	119.6 (3)
N3—Cu—O1	86.78 (10)	C20—C19—H19A	120.2
N1—Cu—O2	93.42 (10)	C18—C19—H19A	120.2
N4^i^—Cu—O2	83.60 (11)	N4—C20—C19	122.4 (3)
N2^i^—Cu—O2	87.10 (11)	N4—C20—H20A	118.8
N3—Cu—O2	99.53 (10)	C19—C20—H20A	118.8
O1—Cu—O2	170.35 (8)	C20—N4—C16	117.9 (3)
C5—N1—C1	116.8 (3)	C20—N4—Cu^ii^	120.3 (2)
C5—N1—Cu	122.5 (2)	C16—N4—Cu^ii^	121.7 (2)
C1—N1—Cu	120.6 (2)	Cu—O1—HW1	116.8
N1—C1—C2	123.0 (3)	Cu—O1—HW2	114.0
N1—C1—H1A	118.5	HW1—O1—HW2	128.7
C2—C1—H1A	118.5	Cu—O2—H2C	111.5
C3—C2—C1	119.5 (4)	Cu—O2—H2D	119.5
C3—C2—H2A	120.3	H2C—O2—H2D	116.1
C1—C2—H2A	120.3	H3C—O3—H3D	108.6
C2—C3—C4	118.1 (3)	C27—O5—H5C	110.2
C2—C3—S1	116.7 (3)	O4—C27—O5	121.5 (4)
C4—C3—S1	125.1 (3)	O4—C27—C21	119.6 (4)
C5—C4—C3	118.7 (3)	O5—C27—C21	118.9 (4)
C5—C4—H4A	120.6	C26—C21—C22	118.5 (3)
C3—C4—H4A	120.6	C26—C21—C27	113.5 (4)
N1—C5—C4	123.8 (3)	C22—C21—C27	128.1 (3)
N1—C5—H5A	118.1	C23—C22—C21	119.0 (3)
C4—C5—H5A	118.1	C23—C22—C28	114.2 (4)
C3—S1—S2	106.16 (13)	C21—C22—C28	126.8 (4)
C8—S2—S1	105.40 (14)	C22—C23—C24	121.5 (4)
N2—C6—C7	123.4 (3)	C22—C23—H23A	119.2
N2—C6—H6A	118.3	C24—C23—H23A	119.2
C7—C6—H6A	118.3	C25—C24—C23	119.6 (4)
C6—C7—C8	118.6 (3)	C25—C24—H24A	120.2
C6—C7—H7A	120.7	C23—C24—H24A	120.2
C8—C7—H7A	120.7	C26—C25—C24	119.6 (4)
C9—C8—C7	119.2 (3)	C26—C25—H25A	120.2
C9—C8—S2	125.4 (2)	C24—C25—H25A	120.2
C7—C8—S2	115.3 (3)	C25—C26—C21	121.7 (4)
C10—C9—C8	118.4 (3)	C25—C26—H26A	119.1
C10—C9—H9A	120.8	C21—C26—H26A	119.1
C8—C9—H9A	120.8	O7—C28—O6	123.2 (5)
N2—C10—C9	123.7 (3)	O7—C28—C22	118.7 (5)
N2—C10—H10A	118.1	O6—C28—C22	118.0 (5)
C9—C10—H10A	118.1	C35—O8—H8C	111.4
C6—N2—C10	116.6 (3)	O9—C35—O8	119.2 (4)
C6—N2—Cu^ii^	119.3 (2)	O9—C35—C29	120.4 (3)
C10—N2—Cu^ii^	123.7 (2)	O8—C35—C29	120.3 (4)
C15—N3—C11	116.8 (3)	C34—C29—C30	117.9 (3)
C15—N3—Cu	118.1 (2)	C34—C29—C35	114.3 (3)
C11—N3—Cu	124.6 (2)	C30—C29—C35	127.8 (3)
N3—C11—C12	123.0 (3)	C31—C30—C29	118.5 (3)
N3—C11—H11A	118.5	C31—C30—C36	112.8 (3)
C12—C11—H11A	118.5	C29—C30—C36	128.7 (3)
C13—C12—C11	119.7 (3)	C32—C31—C30	122.5 (4)
C13—C12—H12A	120.2	C32—C31—H31A	118.8
C11—C12—H12A	120.2	C30—C31—H31A	118.8
C12—C13—C14	117.9 (3)	C33—C32—C31	118.7 (4)
C12—C13—S3	126.4 (2)	C33—C32—H32A	120.6
C14—C13—S3	115.7 (3)	C31—C32—H32A	120.6
C15—C14—C13	118.3 (3)	C34—C33—C32	119.8 (4)
C15—C14—H14A	120.9	C34—C33—H33A	120.1
C13—C14—H14A	120.9	C32—C33—H33A	120.1
N3—C15—C14	124.3 (3)	C33—C34—C29	122.6 (4)
N3—C15—H15A	117.8	C33—C34—H34A	118.7
C14—C15—H15A	117.8	C29—C34—H34A	118.7
C13—S3—S4	105.02 (13)	O10—C36—O11	123.0 (4)
C18—S4—S3	106.42 (13)	O10—C36—C30	119.3 (4)
N4—C16—C17	122.9 (3)	O11—C36—C30	117.7 (4)

Symmetry codes: (i) *x*, *y*+1, *z*; (ii) *x*, *y*−1, *z*.

### Hydrogen-bond geometry (Å, °)

**Table:**

*D*—H···*A*	*D*—H	H···*A*	*D*···*A*	*D*—H···*A*
O1—HW1···O7	0.81	2.51	3.118 (6)	133
O1—HW2···O3^iii^	0.81	1.92	2.658 (4)	153
O2—H2C···O7^iv^	0.75	2.13	2.878 (4)	174
O2—H2D···O9	0.80	2.05	2.841 (4)	171
O3—H3C···O4^iv^	0.81	2.02	2.800 (4)	163
O3—H3D···O11	0.76	2.03	2.784 (5)	172
O5—H5C···O6	0.85	1.51	2.358 (5)	178
O8—H8C···O11	0.87	1.50	2.367 (4)	178

Symmetry codes:  (iii) −*x*+1/2, *y*+1/2, *z*−1/2; (iv) −*x*+1, −*y*+1, *z*+1/2.

## References

[bb1] Flack, H. D. (1983). *Acta Cryst.* A**39**, 876–881.

[bb2] Higashi, T. (1995). *ABSCOR* Rigaku Corporation, Tokyo, Japan.

[bb3] Horikoshi, R. & Mochida, T. (2006). *Coord. Chem. Rev.* **250**, 2595–2609.

[bb4] Luo, J., Hong, M., Wang, R., Yuan, D., Cao, R., Han, L., Xu, Y. & Lin, Z. (2003). *Eur. J. Inorg. Chem.* pp. 3623–3632.

[bb5] Manna, S. C., Konar, S., Zangrando, E., Drew, M. G. B., Ribas, J. & Chaudhuri, N. R. (2005). *Eur. J. Inorg. Chem.* pp. 1751–1758.

[bb6] Manna, S. C., Ribas, J., Zangrando, E. & Chaudhuri, N. R. (2007). *Polyhedron*, **26**, 4923–4928.

[bb7] Rigaku (1998). *RAPID-AUTO* Rigaku Corporation, Tokyo, Japan.

[bb8] Rigaku/MSC (2004). *CrystalStructure* Rigaku/MSC Inc., The Woodlands, Texas, USA.

[bb9] Sheldrick, G. M. (2008). *Acta Cryst.* A**64**, 112–122. 10.1107/S0108767307043930 18156677

